# Longitudinal Pathways From Childhood Maltreatment to NSSI in Middle School Students With Depressive Symptoms: A Latent Change Score Analysis

**DOI:** 10.1155/da/6659147

**Published:** 2025-12-17

**Authors:** Jinwen Li, Minjie Zheng, Rong Bai, Dini Xue, Xia Liu

**Affiliations:** ^1^ Institute of Developmental Psychology, Faculty of Psychology, Beijing Normal University, No. 19 Xinjiekouwai Street, Beijing, 100875, China, bnu.edu.cn

**Keywords:** childhood maltreatment, emotional reactivity, latent change score model, non-suicidal self-injury, self-esteem

## Abstract

**Purpose:**

Adolescents with depressive symptoms are particularly vulnerable and at higher risk for developing co‐occurring mental health issues, such as non‐suicidal self‐injury (NSSI). This study focused on adolescents exhibiting persistent depressive symptoms across three time points and investigated how childhood maltreatment influences NSSI and its changes over time, along with the underlying mechanisms.

**Methods:**

From a larger sample of 3981 students, 317 adolescents with persistent depressive symptoms (*M*_age_ = 13.20, SD = 0.82) were identified, and a control group of 317 non‐depressed adolescents was selected via propensity score matching. Latent change score (LCS) modeling and longitudinal mediation models were employed to examine the mechanisms linking childhood maltreatment to NSSI and its changes.

**Results:**

The univariate LCS model revealed an increasing trend in NSSI behaviors among adolescents with depressive symptoms over the measurement period. T1 childhood maltreatment indirectly influenced T2 NSSI through T2 self‐esteem and T2 emotional reactivity. Additionally, T1 childhood maltreatment significantly predicted changes in NSSI through T2 emotional reactivity.

**Conclusions:**

This study underscores the importance of addressing NSSI in adolescents with depressive symptoms and highlights the necessity of screening for childhood maltreatment histories in this population. The findings provide a foundation for future prevention and intervention efforts, suggesting that enhancing self‐esteem and reducing emotional reactivity could mitigate the impact of childhood maltreatment on NSSI in depressed adolescents.

## 1. Introduction

Among the myriad mental health challenges emerging in adolescents with depressive symptoms, non‐suicidal self‐injury (NSSI) is a critical psychopathological concern. NSSI is defined as the deliberate, self‐inflicted destruction of body tissue without conscious suicidal intent and for purposes not culturally sanctioned, such as cutting, burning, or head‐banging [1]. Although not lethal, research has consistently shown that NSSI is closely associated with numerous adverse outcomes, including functional impairment, diminished quality of life, co‐occurring mental health problems, future self‐harm, and substance abuse [2, 3]. Individuals with mood disorders, such as depression, are significantly more likely to engage in NSSI than their peers in the general population [4, 5]. Indeed, while the prevalence of NSSI among community samples of adolescents is ~22% [6], this rate escalates to 52% among adolescents diagnosed with depression and other mood disorders [7]. Critically, the co‐occurrence of NSSI and depression signals a more severe clinical profile, associated with consequences such as executive dysfunction [8], hypothalamic–pituitary–adrenal axis dysregulation [9], and an increased risk for severe psychiatric outcomes, including suicide [10, 11]. However, despite the high prevalence and potential for harm associated with NSSI, research focusing on its specific risk factors and mechanisms within depressed populations remains relatively limited [12].

Previous theoretical and empirical studies have established childhood maltreatment as a significant factor influencing NSSI in both clinical and non‐clinical populations [13–16]. For instance, the temporal framework of NSSI suggests that distal traits such as childhood maltreatment may heighten vulnerability to NSSI by altering the hypothalamic–pituitary–adrenal axis, brain structure and function, and inflammatory pathways [17]. Longitudinal studies, in particular, have confirmed the prospective association between childhood maltreatment and subsequent NSSI [18–20]. However, these studies have predominantly focused on how childhood maltreatment at an earlier time point predicts the level of NSSI at a later time point, leaving a gap in understanding how childhood maltreatment predicts within‐person changes in NSSI, particularly among youth with depressive symptoms. Latent change score modeling is a powerful technique for such dynamic processes [21]. Compared to other models of change, such as latent growth models, LCS modeling can directly model the “amount of change” between two time points to investigate micro‐level processes and mechanisms, making it the ideal approach to explore within‐person changes in NSSI and elucidate how childhood maltreatment shapes this developmental course in depressed adolescents.

Emotion‐related pathways may play a critical role in the underlying mechanism through which childhood maltreatment influences NSSI [22, 23]. Emotional reactivity, defined as the intensity, depth, and persistence of emotional responses to adverse events [24], may help elucidate the mechanisms of NSSI as proposed by the interpersonal model. This model suggests that harmful interpersonal experiences, such as childhood maltreatment, can trigger emotional reactivity, which in turn increases the likelihood of NSSI [25]. Supporting this, previous research has confirmed that emotional reactivity mediates the relationship between negative experiences like stress and NSSI in university student populations [26, 27]. However, it remains unclear whether this mediational pathway is valid for adolescents with depressive symptoms or if it differs from the mechanism in non‐depressed youth. According to the negative potentiation theory, individuals with depression exhibit heightened emotional reactivity in response to negative stimuli [28, 29], whereas the emotion context‐insensitivity hypothesis [30] and positive attenuation theory [31] emphasize that depression attenuates emotional reactivity. Despite these theoretical discrepancies, all highlight that emotional reactivity in adolescents with depressive symptoms may differ from that of non‐depressed individuals. These findings suggest that the mechanism through which childhood maltreatment influences NSSI via emotional reactivity may be particularly unique in depressed adolescents.

In addition to affecting NSSI through emotional reactivity, childhood maltreatment may also exert an influence through alternative mechanisms. According to the developmental psychopathology model of NSSI, both emotional processes (e.g., emotional reactivity) and internalized representations of the self (e.g., self‐esteem) are mechanisms that can contribute to the development of NSSI behaviors [32]. Previous research has confirmed that childhood maltreatment can influence NSSI through self‐esteem [33, 34], but these studies have primarily focused on the role of self‐esteem without concurrently considering the mediating influence of emotional‐related factors. In fact, when both self‐esteem and emotional reactivity are considered together, which more closely reflects real‐world conditions, the mechanisms by which childhood maltreatment affects NSSI may differ. For example, one study found that when examining a single mechanism variable, childhood maltreatment significantly influenced NSSI through three pathways, including post‐traumatic stress symptoms, but when multiple mechanisms were examined simultaneously, only post‐traumatic stress symptoms mediated the relationship between childhood maltreatment and NSSI [35]. Therefore, the present study investigates how this dual‐pathway mechanism may mediate the relationship between childhood maltreatment and NSSI, offering potential therapeutic interventions for NSSI in depressed adolescents from both emotional and self‐related perspectives.

Focusing on NSSI, a behavior with high prevalence and severe consequences among depressed adolescents, the present study employed a three‐wave longitudinal design with the following aims: (1) to examine the predictive effect of childhood maltreatment on NSSI in adolescents with depressive symptoms and to determine if this association differs from that in non‐depressed adolescents, (2) to explore a dual‐pathway mechanism by investigating the mediating roles of both emotional reactivity and self‐esteem in the relationship between childhood maltreatment and NSSI, and (3) to analyze the impact of childhood maltreatment on the within‐person changes in NSSI over time.

## 2. Methods

### 2.1. Participants and Procedures

This analysis was based on three consecutive assessments (December 2021, June 2022, and March 2023) of an ongoing longitudinal study focusing on adolescents’ emotional and behavioral development (AEBD study). Due to the COVID‐19 epidemic, an online platform (Wenjuanxing) was used to collect data. After signing informed consent, students completed the questionnaire online through computers in a microcomputer classroom under the guidance of trained research assistants. Students who completed the questionnaire received a gift following submission. The Research Ethics Committee of the authors’ university approved all procedures.

A total of 3981 students from three public junior high schools in Shanxi Province participated in the AEBD study. For the present analysis, we identified adolescents who exhibited depressive symptoms at T1, T2, and T3. Depressive symptoms were assessed using the 10‐item Center for Epidemiological Studies Depression Scale (CESD‐10) [36], which has been widely used and validated [37]. Following the established standard cutoff score of CESD‐10 ≥10 [38], 331 adolescents met the inclusion criteria at all three waves. Of these, 14 were excluded due to failing all attention checks (e.g., “Please respond with ‘Disagree’ for this item”) or excessively short response times (average response time per item <2 s) [39, 40]. Additionally, individuals with severe intellectual disabilities or severe suicidal tendencies were excluded. The final sample comprised 317 participants (41.0% male), with a mean age of 13.20 years (SD = 0.82) at T1, ranging from 11 to 15 years. To compare pathway differences between groups, we then selected a control group. Using propensity score matching to balance key demographic characteristics (e.g., age, gender, and family socioeconomic status [SES]), 317 non‐depressed adolescents were selected from the original cohort to form the non‐depressed group (*M*_age_ = 12.77, SD = 0.75).

### 2.2. Measures

#### 2.2.1. Childhood Maltreatment

Childhood maltreatment was assessed using the Chinese version of the Childhood Trauma Questionnaire‐Short Form (CTQ‐SF) [41]. A total of 20 items were included in the current study to assess experiences of physical abuse, emotional abuse, physical neglect, and emotional neglect before the age of 12. Items were scored on a 5‐point scale, ranging from 1 (never) to 5 (always). After reverse‐scoring the negatively worded items, the average score was calculated, with higher scores indicating more severe abuse and neglect in childhood. The CTQ‐SF has been widely used to measure childhood maltreatment in Chinese adolescents and has demonstrated good reliability and validity [42, 43]. In the present study, the Cronbach’s *α* for the childhood maltreatment scale was 0.89.

#### 2.2.2. Emotional Reactivity

Emotional reactivity was assessed using the Emotional Reactivity Scale (ERS) [24], a 21‐item scale meticulously tailored to quantify emotion sensitivity, intensity, and persistence. Items were scored on a 5‐point scale, ranging from 1 (completely disagree) to 5 (completely agree). A higher average score on the ERS indicates higher levels of emotional reactivity. The ERS has been well‐validated for use with Chinese adolescents [44]. The scale also showed excellent internal consistency in this study, with a Cronbach’s *α* of 0.96.

#### 2.2.3. Self‐Esteem

The 10‐item Rosenberg [45] Self‐Esteem Scale was used to assess the self‐esteem. Items were rated on a 5‐point scale from 1 (completely disagree) to 5 (completely agree). After reverse‐scoring the negatively worded items, the average score was calculated, with higher scores indicating higher levels of self‐esteem. This scale has been widely used for measuring self‐esteem in Chinese adolescents [46]. In the current study, the Cronbach’s *α* for the scale was 0.85.

#### 2.2.4. NSSI

NSSI behaviors in the previous 6 months were assessed using a modified Chinese version of the Deliberate Self‐harm Inventory (DSHI) [47]. The DSHI is a self‐report measure that asks participants to report the frequency of nine common NSSI behaviors, ranging from 0 (none) to 6 (five times or more). A higher average score on all items indicated a greater frequency of NSSI behaviors. This inventory has been widely used and validated in adolescent populations [48]. In the current study, the scale demonstrated good internal consistency, with Cronbach’s *α*s of 0.91 at T2 and 0.88 at T3.

#### 2.2.5. Sociodemographic Information

Adolescent participants provided information regarding age, gender, and SES. SES was assessed using the well‐established MacArthur Scale [49]. A 10‐step class ladder was presented, and participants were informed that a higher step indicates a higher SES rank. A higher score indicated a higher subjective SES.

### 2.3. Statistical Analysis

All primary hypotheses were tested within a structural equation modeling (SEM) framework using Mplus 8.3, with descriptive statistics generated by the bruceR package (Bao, 2024) in R (Version 4.4.1). Our analytical strategy proceeded in three main stages. First, we established the longitudinal foundation of our model by specifying a univariate latent change score (LCS) model [50], aiming to formally quantify the within‐person changes in NSSI between assessment points. Subsequently, the LCS framework was expanded into a dual‐pathway mediation model to test our primary hypotheses, examining whether childhood maltreatment influences both the initial level and subsequent change in NSSI through the mediating roles of emotional reactivity and self‐esteem among adolescents with depressive symptoms. Finally, we conducted a multi‐group analysis comparing the model across depressive symptom status and gender. Using the MODEL CONSTRAINT command, we directly tested for group differences in key structural paths. All path coefficients and indirect effects were tested for significance using bias‐corrected 95% confidence intervals derived from 5000 bootstrap samples, with an interval not containing zero indicating a significant effect.

Full information maximum likelihood (FIML) estimation was utilized to handle sporadic missing data, ensuring maximal utilization of the available sample. Model fit was evaluated by multiple indices, with CFI ≥ 0.90, RMSEA ≤ 0.08, and SRMR ≤ 0.08 indicating an acceptable model.

## 3. Results

### 3.1. Descriptive Analysis of Main Variables

The prevalence of NSSI among adolescents with depressive symptoms was 67.8% at T2 and 63.1% at T3. Notably, 42.6% of adolescents met the criteria for repetitive NSSI at T2 [51], and 41.6% met the criteria at T3. As shown in Table 1, compared to adolescents without depressive symptoms, those with depressive symptoms experienced more childhood maltreatment (*t* = –13.89, *p* < 0.001), had lower levels of self‐esteem (*t* = 27.95, *p* < 0.001), exhibited higher levels of emotional reactivity (*t* = –23.92, *p* < 0.001), and reported higher levels of NSSI (all *p* < 0.001).

**Table 1:** Descriptive statistics of the main variables among adolescents with and without depressive symptoms.

Variable	Adolescents with depressive symptoms			Adolescents without depressive symptoms			*t*	*p*
	*M*	SD	Prevalence	*M*	SD	Prevalence		
1. T1 childhood maltreatment	1.61	0.56	—	1.15	0.18	—	−13.89	<0.001
2. T2 self‐esteem	2.77	0.69	—	4.22	0.61	—	27.95	<0.001
3. T2 emotional reactivity	3.19	0.92	—	1.67	0.66	—	−23.92	<0.001
4. T2 NSSI	0.93	1.31	67.8%	0.07	0.24	15.8%	−11.41	<0.001
5. T3 NSSI	0.80	1.16	63.1%	0.05	0.16	13.2%	−11.43	<0.001

*Note*: Adolescents who scored below the standard cutoff score of 10 on the CESD‐10 at all three time points were classified as non‐depressed. Using propensity score matching, 317 non‐depressed individuals were selected and included in the non‐depressed group; NSSI, non‐suicidal self‐injury.

**Figure 1:**
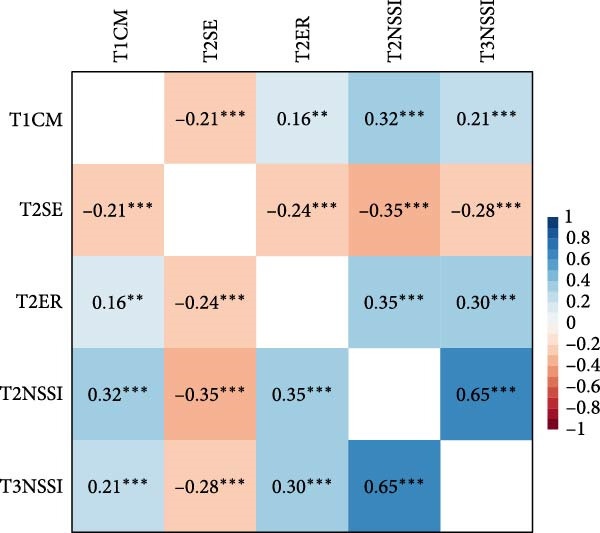
Correlation matrix of main variables among adolescents with depressive symptoms.  ^∗^ *p* < 0.05.  ^∗∗^ *p* < 0.01.  ^∗∗∗^ *p* < 0.001. T1CM, childhood maltreatment at T1; T2SE, self‐esteem at T2; T2ER, emotional reactivity at T2; NSSI, non‐suicidal self‐Injury.

Among adolescents with depressive symptoms, T1 childhood maltreatment was negatively correlated with T2 self‐esteem (*r* = –0.21, *p* < 0.001) and positively correlated with T2 emotional reactivity (*r* = 0.16, *p* = 0.005), T2 NSSI (*r* = 0.32, *p* < 0.001), and T3 NSSI (*r* = 0.21, *p* < 0.001). T2 self‐esteem was negatively associated with T2 emotional reactivity (*r* = –0.24, *p* < 0.001), T2 NSSI (*r* = –0.35, *p* < 0.001), and T3 NSSI (*r* = –0.28, *p* < 0.001). T2 emotional reactivity was positively correlated with T2 NSSI (*r* = 0.35, *p* < 0.001) and T3 NSSI (*r* = 0.30, *p* < 0.001). T2 NSSI showed positive correlations with T3 NSSI (*r* = 0.65, *p* < 0.001). Detailed correlation coefficients are shown in Figure 1.

### 3.2. The Mechanisms Linking Childhood Maltreatment to NSSI and Its Changes

Among adolescents with depressive symptoms, the univariate LCS showed significant developmental increases in NSSI during the measurements (mean = 0.275; 95% CI [0.184, 0.373]; *p* < 0.001). Also, the variances in latent change scores of NSSI were significantly different from zero (variance = 0.804; 95% CI [0.629, 1.009]; *p* < 0.001).

**Figure 2:**
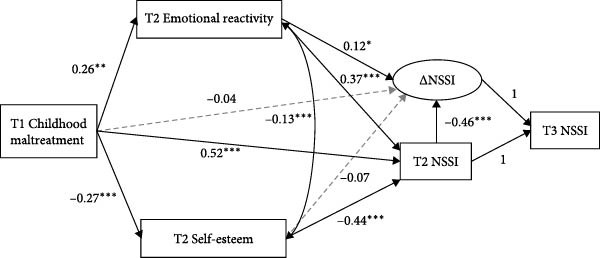
The mechanisms linking childhood maltreatment to NSSI and its changes among adolescents with depressive symptoms. All paths are unstandardized; gender, age, and socioeconomic status were included as covariates but are not presented in the figure for simplicity. NSSI: non‐suicidal self‐injury; *Δ*NSSI: latent difference score representing the change in NSSI from T2 to T3. Solid lines represent significant paths; dashed lines represent non‐significant paths.  ^∗^ *p* < 0.05,  ^∗∗^ *p* < 0.01,  ^∗∗∗^ *p* < 0.001.

The hypothesized mediation model among the depressed youth fit the data well, with CFI = 0.964, SRMR = 0.045, and RMSEA = 0.077. As shown in Figure 2, T1 childhood maltreatment negatively predicted T2 self‐esteem (*B* = –0.27, 95% CI [–0.412, –0.112]) and positively predicted T2 emotional reactivity (*B* = 0.26, 95% CI [0.059, 0.440]) and T2 NSSI (*B* = 0.52, 95% CI [0.222, 0.807]). T2 Emotional reactivity positively predicted T2 NSSI (*B* = 0.37, 95% CI [0.208, 0.535]), while T2 self‐esteem negatively predicted T2 NSSI (*B* = –0.44, 95% CI [–0.646, –0.238]). Furthermore, T1 childhood maltreatment significantly predicted T2 NSSI through both T2 emotional reactivity (*B* = 0.10, 95% CI [0.027, 0.193]) and T2 self‐esteem (*B* = 0.12, 95% CI [0.045, 0.226]).

T2 self‐esteem did not significantly predict *Δ*NSSI (*B* = –0.07, 95% CI [–0.231, 0.100]), whereas T2 emotional reactivity significantly positively predicted *Δ*NSSI (*B* = 0.12, 95% CI [0.012, 0.230]), indicating that higher emotional reactivity was associated with faster increases in NSSI among adolescents with depressive symptoms. The direct effect of T1 childhood maltreatment on *Δ*NSSI was not significant (*B* = −0.04, 95% CI [–0.280, 0.212]), and the mediating effect of T2 self‐esteem on *Δ*NSSI was also insignificant (*B* = 0.02, 95% CI [–0.024, 0.074]). However, T1 childhood maltreatment could predict *Δ*NSSI through T2 emotional reactivity (*B* = 0.03, 95% CI [0.003, 0.078]).

**Figure 3:**
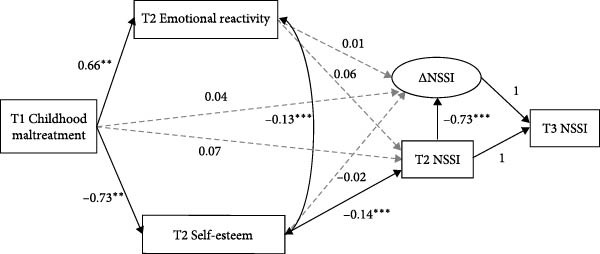
The mechanisms linking childhood maltreatment to NSSI and its changes among adolescents without depressive symptoms. All paths are unstandardized; gender, age, and socioeconomic status were included as covariates but are not presented in the figure for simplicity. NSSI: non‐suicidal self‐injury; *Δ*NSSI: Latent difference score representing the change in NSSI from T2 to T3. Solid lines represent significant paths; dashed lines represent non‐significant paths.  ^∗^ *p* < 0.05,  ^∗∗^ *p* < 0.01,  ^∗∗∗^ *p* < 0.001.

The hypothesized mediation model among non‐depressed youth also demonstrated an acceptable model fit, with CFI = 0.938, SRMR = 0.045, and RMSEA = 0.084. As depicted in Figure 3, for adolescents without depressive symptoms, T1 childhood maltreatment significantly predicted T2 NSSI through the mediating pathway of T2 self‐esteem (*B* = 0.10, 95% CI [0.039, 0.217]), but the pathway through T2 emotional reactivity was not significant (*B* = 0.04, 95% CI [0.000, 0.099]). Furthermore, for the non‐depressed group, neither self‐esteem (*B* = 0.01, 95% CI [–0.012, 0.043]) nor emotional reactivity (*B* = 0.01, 95% CI [–0.011, 0.038]) mediated the pathway from childhood maltreatment to *Δ*NSSI.

A multi‐group analysis was conducted to directly compare the structural pathways between adolescents with and without depressive symptoms. The results showed that while the overall indirect effects did not differ significantly between the two groups, several constituent direct paths of the model varied. As presented in Table S1, Wald tests confirmed that for the depressed group, the predictive paths from T1 childhood maltreatment, T2 emotional reactivity, and T2 self‐esteem to T2 NSSI were all significantly stronger than those for the non‐depressed group.

### 3.3. Sensitivity Analysis

To probe the robustness of the mediating pathways from childhood maltreatment to NSSI among the depressed group, we conducted a series of sensitivity analyses. First, a multi‐group analysis was performed to determine whether the mediational mechanism differed by gender. The results indicated that none of the direct or indirect path coefficients differed significantly between genders, as the 95% confidence intervals for all parameter differences included 0. This finding suggests that the mediating roles of self‐esteem and emotional reactivity are comparable for male and female adolescents. Detailed results are available in Table S2.

Second, to ensure our findings were not confounded by potential moderation effects, we conducted a sensitivity analysis by incorporating all two–way interaction terms among the primary predictor (T1 childhood maltreatment) and covariates (gender, age, and SES) into the mediation model. The results remained robust. Specifically, the indirect effects of T1 childhood maltreatment on T2 NSSI through both T2 self‐esteem (*B* = 0.12, 95% CI [0.045, 0.224]) and T2 emotional reactivity (*B* = 0.10, 95% CI [0.028, 0.201]) were still significant. The indirect effect on *Δ*NSSI via T2 emotional reactivity also held significant (*B* = 0.03, 95% CI [0.002, 0.081]), whereas the pathway via T2 self‐esteem did not (*B* = 0.02, 95% CI [–0.019, 0.079]). None of the newly added interaction terms were significant (*p* > 0.05), supporting the robustness of our primary mediation model.

## 4. Discussion

While extensive research has documented the link between childhood maltreatment and NSSI, the specific pathways within the high‐risk population of depressed youth remain less understood. The present study found that the prevalence of NSSI among adolescents with depressive symptoms remained above 60% at both measurement points, with the rate of repetitive NSSI surpassing 40%. This rate of repetition is clinically alarming, as prior work suggests nearly half of those with repetitive NSSI meet the diagnostic criteria for DSM‐5 NSSI Disorder [52]. Focusing on the etiological pathways of NSSI in these depressed youth, we found that childhood maltreatment predicted NSSI through both self‐esteem and emotional reactivity but only predicted the change in NSSI through emotional reactivity within this group.

Although the link between childhood maltreatment and NSSI is well‐documented, much of the existing evidence is cross‐sectional, limiting causal inference [13]. Our prospective findings establish the temporal precedence of maltreatment, enhancing the plausibility of a causal link to adolescent NSSI. Crucially, we found the direct effect of childhood maltreatment on NSSI in the depressed group was significantly stronger than in the non‐depressed group. The heightened risk for NSSI in depressed adolescents is likely rooted in a heightened neurobiological vulnerability. For instance, childhood adversity has been linked to HPA axis dysregulation, aberrant cortisol levels, and altered neural function (e.g., changes in the amplitude of low‐frequency fluctuations) in depressed individuals [53], which may potentiate the risk for NSSI.

The bootstrapping results indicate that emotional reactivity mediates the relationship between childhood maltreatment and NSSI among adolescents with depressive symptoms, which is consistent with previous findings in general university student samples [26, 27]. Moving beyond this finding, by directly comparing pathways between depressed and non‐depressed groups, our study provides evidence to inform the ongoing debate about emotional reactivity in depression. Existing frameworks are divided on whether depression heightens or attenuates emotional reactivity [28–30]. Our path analysis revealed that while the effect of childhood maltreatment on emotional reactivity did not differ between depressed and non‐depressed adolescents, the predictive effect of emotional reactivity on NSSI was significantly stronger in the depressed group, supporting the negative potentiation theory. Previous research has similarly indicated that depressive symptoms can amplify adolescents’ emotional reactivity to negative interpersonal events, such as daily perceptions of peer rejection [54]. This implies that, even when exposed to comparable negative stressors, adolescents with depressive symptoms are likely to experience heightened emotional responses, thereby rendering them more prone to engaging in NSSI.

It is worth noting that, compared to previous studies that examined the impact of childhood maltreatment on NSSI through emotional pathways [18, 55], this study further focused on within‐person changes in NSSI. Childhood maltreatment was found to indirectly contribute to the increase in NSSI behaviors among depressed adolescents by influencing emotional reactivity, thereby expanding the findings in this area. Moreover, emotional reactivity appears to be a unique mechanism underlying NSSI in depressed adolescents. This finding is particularly valuable, as it carries significant implications for guiding interventions. For individuals who have already exhibited depressive symptoms, screening for childhood maltreatment experiences and implementing tailored interventions to reduce emotional reactivity, such as Mindfulness‐Based Cognitive Therapy [56], may help mitigate NSSI.

In addition to impacting NSSI through emotional reactivity, the current study revealed that childhood maltreatment also contributes to an increased incidence of NSSI by undermining self‐esteem. This finding can be understood in two ways. First, negative self‐perception and self‐evaluation are prominent characteristics among adolescents with depression [57]. As such, when faced with childhood maltreatment, depressed adolescents may be more likely to self‐blame, leading to lower self‐esteem and a higher risk of NSSI. Second, recent research has shown that individuals with depression often struggle to revise negative beliefs in response to positive information [58]. For depressed adolescents with a history of childhood maltreatment, even though they may receive support from friends, teachers, or family, they are unable to update their negative beliefs with this supportive input or positive information. This difficulty in revising negative beliefs allows low self‐esteem to persist, leading to more instances of NSSI. In contrast to previous studies that focused on single pathways, our research demonstrates that both emotional and self‐esteem pathways co‐exist in depressed adolescents, whereas the self‐esteem pathway appears to be primary in non‐depressed youth. These findings highlight the importance of developing subgroup‐specific intervention strategies for adolescents with and without depressive symptoms. For adolescents with depressive symptoms, simultaneous intervention on both emotional reactivity and self‐esteem may be particularly effective in reducing NSSI. In contrast, among adolescents without depressive symptoms, programs that primarily focus on enhancing self‐worth, such as self‐affirmation intervention [59], positive psychology programs [60], or cognitive–behavioral strategies targeting maladaptive self‐beliefs [61], may be more appropriate.

Interestingly, the present study found that while self‐esteem mediated the relationship between childhood maltreatment and T2 NSSI, providing support for the developmental psychopathology model of NSSI [32], it did not significantly predict *Δ*NSSI. This discrepancy extends our understanding of the developmental psychopathology model of NSSI and may be explained by distinguishing between between‐person effects (T2 NSSI) and within‐person effects (*Δ*NSSI). Although prior research has demonstrated that, at the between‐person level, self‐esteem negatively predicts NSSI severity, previous studies have also shown that within‐person dynamics may diverge from between‐person associations [62]. At the within‐person level, it is plausible that for an adolescent whose NSSI level is already relatively high, a further decrease in self‐esteem may not necessarily trigger a corresponding increase in NSSI. In such cases of a ceiling or stabilization effect, self‐esteem would not emerge as a significant predictor of change, which is consistent with our findings.

Several limitations should be considered when interpreting these findings. First, the “depressed adolescents” in this study were identified based on self‐reported depressive symptoms in a community sample drawn from three Chinese middle schools within a narrow age range (11–15 years), which may limit the representativeness of the sample. Therefore, caution should be exercised when generalizing these results to clinically diagnosed populations or to adolescents in other regions or age groups (e.g., senior high school students). Second, the reliance on self‐report measures for all key variables may introduce recall bias, response bias, or social desirability effects, limiting causal inference. Future research could incorporate multi‐source and multi‐modal data to provide a more comprehensive understanding. For instance, investigating the underlying physiological and psychological mechanisms by integrating biomarkers, such as DNA methylation [63], may further elucidate the link between childhood maltreatment and NSSI. Third, while we focused on the dynamic changes in NSSI, our mediators were only measured at a single time point and were not modeled longitudinally. This prevents us from establishing the complete temporal precedence of the mediational chain (i.e., that change in the mediator precedes change in the outcome). Future research is needed to examine how childhood maltreatment influences the change of NSSI through the corresponding developmental changes in self‐esteem and emotional reactivity. Additionally, our study’s 6‐month follow‐up interval may not capture other temporal patterns, such as seasonal variations in self‐injury, which could be explored with different assessment schedules in subsequent research. Finally, this study did not account for other potential confounding variables, such as comorbid anxiety, which could potentially confound the mediating effects (e.g., anxiety may amplify emotional reactivity). Future research should include and control for such comorbidities to isolate the specific mechanisms more precisely.

## 5. Conclusions

Our research highlights the alarming prevalence of NSSI among adolescents with depressive symptoms, underscoring the urgent need for targeted interventions. Given the limited resources for intervention, prioritizing vulnerable or high‐risk groups, such as depressed adolescents with childhood maltreatment experiences, should be considered a strategic approach when developing NSSI prevention and intervention programs. Additionally, our study identified a dual‐pathway mechanism linking childhood maltreatment to NSSI in adolescents with depressive symptoms, offering valuable insights for future intervention strategies that address both emotional and self‐related factors. Furthermore, using latent change score modeling, we examined the development and changes of NSSI in adolescents with depressive symptoms and found that childhood maltreatment influences NSSI changes through emotional reactivity. This finding reinforces the role of emotional reactivity from a within‐person change perspective. Taken together, our results suggest that screening for childhood maltreatment experiences in adolescents with depressive symptoms could enhance the specificity of subsequent interventions. Interventions targeting self‐esteem and emotional reactivity—particularly emotional reactivity—may offer promising avenues for interrupting the development and progression of NSSI.

## Conflicts of Interest

The authors declare no conflicts of interest.

## Funding

This work was supported by the National Natural Science Foundation of China, 32471115.

## Supporting Information

Additional supporting information can be found online in the Supporting Information section.

## Supporting information


**Supporting Information** Table S1: Comparison of model pathways between depressed and non‐depressed groups. Table S2: Comparison of model pathways between males and females in the depressed group.

## Data Availability

The data that support the findings of this study are available from the corresponding author upon reasonable request.
