# Supplementary material for: Longitudinal Pathways From Childhood Maltreatment to NSSI in Middle School Students With Depressive Symptoms: A Latent Change Score Analysis
Source: Depress Anxiety. 2025 Dec 17;2025:6659147. doi: 10.1155/da/6659147 (PMC12714111; doi:10.1155/da/6659147)
Supplement: Supplementary file 1 — Supporting Information Table S1: Comparison of model pathways between depressed and non‐depressed groups. Table S2: Comparison of model pathways between males and females in the depressed group. [file DA-2025-6659147-s001.docx]

**Appendix 1.**

**Table S1.** Comparison of Model Pathways Between Depressed and Non-Depressed Groups

| Pathways | Adolescents with depressive symptoms | | |  | Adolescents without depressive symptoms | | |  | Differences in Coefficients | | |
| --- | --- | --- | --- | --- | --- | --- | --- | --- | --- | --- | --- |
|  | *B* | *p* | 95CI% |  | *B* | *p* | 95CI% |  | *ΔB* | *p* | 95CI% |
| a1: T1CM→ T2 SE | **–0.267** | **<0.001** | **[–0.412, –0.112]** |  | **–0.723** | **0.002** | **[–1.170, –0.259]** |  | 0.456 | 0.065 | [–0.030, 0.933] |
| a2: T1CM → T2 ER | **0.258** | **0.008** | **[0.059, 0.440]** |  | **0.663** | **0.002** | **[0.256, 1.098]** |  | –0.405 | 0.085 | [–0.878, 0.063] |
| b1: T2 SE → T2NSSI | **–0.438** | **<0.001** | **[–0.646, –0.238]** |  | **–0.144** | **<0.001** | **[–0.227, –0.078]** |  | **–0.294** | **0.008** | **[–0.514, –0.076]** |
| b2: T2 ER → T2NSSI | **0.371** | **<0.001** | **[0.208, 0.535]** |  | 0.056 | 0.085 | [–0.005, 0.121] |  | **0.315** | **0.001** | **[0.139, 0.491]** |
| b3: T2 SE → ΔNSSI | –0.069 | 0.409 | [–0.231, 0.100] |  | –0.015 | 0.386 | [–0.050, 0.020] |  | –0.054 | 0.529 | [–0.219, 0.121] |
| b4: T2 ER → ΔNSSI | **0.115** | **0.039** | **[0.012, 0.230]** |  | 0.014 | 0.437 | [–0.018, 0.050] |  | 0.102 | 0.084 | [–0.010, 0.222] |
| c1: T1CM → T2NSSI | **0.519** | **<0.001** | **[0.222, 0.807]** |  | 0.065 | 0.607 | [–0.138, 0.356] |  | **0.454** | **0.019** | **[0.052, 0.820]** |
| c2: T1CM → ΔNSSI | –0.041 | 0.739 | [–0.280, 0.212] |  | 0.040 | 0.499 | [–0.069, 0.166] |  | –0.081 | 0.553 | [–0.350, 0.189] |

Note: The multi-group model demonstrated good fit indices, with CFI = 0.953, RMSEA = 0.080, and SRMR = 0.045. T1CM, Childhood Maltreatment at T1; T2SE, Self-Esteem at T2; T2ER, Emotional Reactivity at T2; NSSI, Non-Suicidal Self-Injury. ΔB represents the result of the depressed group's coefficient minus the non-depressed group's coefficient. Values in **bold** indicate statistical significance.

**Table S2**. Comparison of Model Pathways Between Males and Females in the Depressed Group

| Pathways | Male | | |  | Female | | |  | Differences in Coefficients | | |
| --- | --- | --- | --- | --- | --- | --- | --- | --- | --- | --- | --- |
|  | *B* | *p* | 95CI% |  | *B* | *p* | 95CI% |  | *ΔB* | *p* | 95CI% |
| a1: T1CM→ T2 SE | **–0.298** | **0.002** | **[–0.514, –0.133]** |  | **–0.245** | **0.028** | **[–0.440, –0.008]** |  | –0.052 | 0.726 | [−0.355, 0.229] |
| a2: T1CM → T2 ER | 0.266 | 0.064 | [–0.029, 0.532] |  | 0.252 | 0.054 | [–0.014, 0.497] |  | 0.013 | 0.945 | [−0.378, 0.385] |
| b1: T2 SE → T2NSSI | **–0.523** | **0.004** | **[–0.891, –0.173]** |  | **–0.386** | **0.002** | **[–0.635, –0.136]** |  | –0.137 | 0.539 | [−0.571, 0.293] |
| b2: T2 ER → T2NSSI | **0.399** | **0.011** | **[0.129, 0.742]** |  | **0.353** | **<0.001** | **[0.156, 0.550]** |  | 0.047 | 0.800 | [−0.284, 0.442] |
| b3: T2 SE → ΔNSSI | –0.051 | 0.729 | [–0.320, 0.261] |  | –0.073 | 0.471 | [–0.284, 0.123] |  | 0.022 | 0.901 | [−0.309, 0.386] |
| b4: T2 ER → ΔNSSI | 0.080 | 0.434 | [–0.107, 0.306] |  | **0.138** | **0.035** | **[0.013, 0.274]** |  | –0.058 | 0.634 | [−0.286, 0.190] |
| c1: T1CM → T2NSSI | **0.516** | **0.027** | **[0.089, 1.012]** |  | **0.526** | **0.006** | **[0.170, 0.914]** |  | –0.010 | 0.973 | [−0.626, 0.591] |
| c2: T1CM → ΔNSSI | –0.016 | 0.939 | [–0.420, 0.391] |  | –0.045 | 0.770 | [–0.323, 0.286] |  | 0.029 | 0.908 | [−0.494, 0.517] |

*Note*. The multi-group model demonstrated good fit indices, with CFI = 0.980, RMSEA = 0.068, and SRMR = 0.044. ΔB represents the result of the male group's coefficient minus the female group's coefficient. Values in **bold** indicate statistical significance.
